# Incidence of Lyme Borreliosis in Europe from National Surveillance Systems (2005–2020)

**DOI:** 10.1089/vbz.2022.0071

**Published:** 2023-04-12

**Authors:** Leah Burn, Thao Mai Phuong Tran, Andreas Pilz, Andrew Vyse, Mark A. Fletcher, Frederick J. Angulo, Bradford D. Gessner, Jennifer C. Moïsi, Luis Jodar, James H. Stark

**Affiliations:** ^1^ P95 Pharmacovigilance & Epidemiology, Princeton, New Jersey, USA.; ^2^ P95 Pharmacovigilance & Epidemiology, Leuven, Belgium.; ^3^ Pfizer Global Medical Affairs, Vaccines, Vienna, Austria.; ^4^ Pfizer Vaccines Medical, Walton Oaks, Surrey, United Kingdom.; ^5^ Pfizer Emerging Markets Medical Affairs, Vaccines, Paris, France.; ^6^ Pfizer Vaccines Medical Development, Scientific and Clinical Affairs, Collegeville, Pennsylvania, USA.; ^7^ Pfizer Medical Development, Scientific and Clinical Affairs, Vaccines, Paris, France.

**Keywords:** Lyme borreliosis, incidence, Europe, epidemiology, surveillance

## Abstract

**Background:**

Lyme borreliosis (LB) is the most common tick-borne disease in Europe. To inform European intervention strategies, including vaccines under development, we conducted a systematic review for LB incidence.

**Methods:**

We searched publicly available surveillance data reporting LB incidence in Europe from 2005 to 2020. Population-based incidence was calculated as the number of reported LB cases per 100,000 population per year (PPY), and high LB risk areas (incidence >10/100,00 PPY for 3 consecutive years) were estimated.

**Results:**

Estimates of LB incidence were available for 25 countries. There was marked heterogeneity in surveillance systems (passive vs. mandatory and sentinel sites vs. national), case definitions (clinical, laboratory, or both), and testing methods, limiting comparison across countries. Twenty-one countries (84%) had passive surveillance; four (Belgium, France, Germany, and Switzerland) used sentinel surveillance systems. Only four countries (Bulgaria, France, Poland, and Romania) used standardized case definitions recommended by European public health institutions. Among all surveillance systems and considering any case definition for the most recently available years, national LB incidences were highest in Estonia, Lithuania, Slovenia, and Switzerland (>100 cases/100,000 PPY), followed by France and Poland (40–80/100,000 PPY), and Finland and Latvia (20–40/100,000 PPY). Incidences were lowest in Belgium, Bulgaria, Croatia, England, Hungary, Ireland, Norway, Portugal, Romania, Russia, Scotland, and Serbia (<20/100,000 PPY). At the subnational level, highest LB incidences (>100/100,000 PPY) were observed in areas of Belgium, Czech Republic, France, Germany, and Poland. Overall, on average 128,888 cases are reported annually. An estimated 202/844 million (24%) persons in Europe reside in areas of high LB incidence and 202/469 million (43.2%) persons reside in areas of high LB incidence among countries with surveillance data.

**Conclusion:**

Our review showed substantial variability in reported LB incidence across and within European countries, with highest incidences reported from the Eastern, Northern (Baltic states and Nordic countries), and Western Europe surveillance systems. Standardization of surveillance systems, including wider implementation of common case definitions, is urgently needed to interpret the range of differences in LB incidence observed across European countries.

## Introduction

Lyme borreliosis (LB) is a tick-borne disease that can affect the skin, nervous system, joints, and heart. LB is due to infection with *Borrelia* spirochetes, which are transmitted to humans by the bite of an infected *Ixodes* spp. tick. LB is the most common vector-borne disease in Europe and is most frequently associated with three *Borrelia* genospecies (*B. afzelii*, *B. garinii*, and *B. burgdorferi sensu stricto)* (Bobe et al, 2021). An estimated 65,000–85,000 LB cases per year are reported in Europe through 2006 (Lindgren and Jaenson, 2006), and a population-weighted estimate of LB incidence is 22 cases per 100,000 person-years in Western Europe (Sykes and Makiello, 2017). Yet incomplete surveillance, missed diagnoses, and use of insensitive laboratory testing methods suggest substantial underreporting (Lindgren and Jaenson, 2006). In recent years, the incidence of LB has increased (Vandekerckhove et al, 2021), possibly due to increased tick populations (changes in climate), expanding reservoirs (land management), evolving human behaviors (more frequent contact with infected ticks), or improved reporting (Janova, 2019).

Clinically, LB can progress through three stages. Stage 1 LB frequently goes unrecognized, although it typically presents as erythema migrans (EM), a skin lesion that appears days to weeks after a bite from an infected tick, which can be accompanied by a range of “flu-like” symptoms (Aucott et al, 2009; Bobe et al, 2021). Stage 2, or early disseminated LB, can affect the nervous system or the heart, presenting as Lyme neuroborreliosis (LNB) or, rarely, as Lyme carditis (LC). Stage 3 or late disseminated disease includes acrodermatitis chronica atrophicans and Lyme arthritis (LA) (Bobe et al, 2021; Steere et al, 2016).

Diagnosis of stages 2 and 3 is confirmed by serologic tests, including enzyme immunoassays, immunofluorescence assays, enzyme-linked immunosorbent assays, Western blot, and other specialized tests, such as detection of intrathecal-specific antibodies in patients with suspected LNB. These tests vary in sensitivity and specificity, limiting comparability between different serodiagnostic tests (Branda and Steere, 2021; Kodym et al, 2018).

LB surveillance systems in Europe were recently reviewed (Blanchard et al, 2022; Nagarajan et al, 2022). These data provide an update to previous reviews (Sykes and Makiello, 2017; Vandekerckhove et al, 2021) that were limited to Western Europe. Surveillance systems variably collect cases of EM, LNB, LA, or any LB manifestation, whether diagnosed by physicians, reported by laboratories, or both. Moreover, surveillance is based on a range of strategies that vary by site (sentinel laboratory or general practice [GP] settings), by scope (regional or national), and by legislation (mandatory vs. nonmandatory reporting), which impedes cross-country comparisons of LB incidence (Van den Wijngaard et al, 2017).

There is currently no widely adopted standardized case definition for LB in Europe. Case definitions used for routine surveillance of LB vary by country according to the combination of criteria applied: clinical (history of a tick bite and spectrum of clinical signs and symptoms) and laboratory (the number and type of diagnostic tests used) (Stanek et al, 2011). In 2011, the European Concerted Action on Lyme Borreliosis (EUCALB) published a series of LB case definitions that incorporate clinical findings and essential laboratory evidence (Table 1**)** (Stanek et al, 2011); however, implementation remains inconsistent. Specific to LNB, the European Commission authorized the European Center for Disease Prevention and Control (ECDC) to develop a standardized case definition to monitor the EU-wide distribution of LNB cases (ECDC, 2018; The Lancet Editorial Board, 2018).

**Table 1.** Standardized Case Definitions of Lyme Borreliosis Available for Europe

EUCALB (Stanek et al, 2011)			
Term	Clinical case definition	Essential laboratory evidence	Supporting laboratory/clinical evidence
EM	Expanding red or bluish-red patch (≥5 cm in diameter),^a^ with or without central clearing. Advancing edge typically distinct, often intensely colored, not markedly elevated.	None	Detection of *Borrelia burgdorferi s.l.* by culture and/or PCR from skin biopsy.
Borrelial lymphocytoma (rare)	Painless bluish-red nodule or plaque, usually on ear lobe, ear helix, nipple, or scrotum; more frequent in children (especially on ear) than in adults.	Seroconversion or positive serology^b^ Histology in unclear cases	Histology. Detection of *B. burgdorferi s.l.* by culture and/or PCR from skin biopsy. Recent or concomitant EM.
Acrodermatitis chronic atrophicans	Long-standing red or bluish-red lesions, usually on the extensor surfaces of extremities. Initial doughy swelling. Lesions eventually become atrophic. Possible skin induration and fibroid nodules over bony prominences.	High level of specific serum IgG antibodies	Histology. Detection of *B. burgdorferi s.l.* by culture and/or PCR from skin biopsy.
LNB	In adults mainly meningoradiculitis, meningitis; rarely encephalitis, myelitis; very rarely cerebral vasculitis. In children, mainly meningitis and facial palsy.	Pleocytosis and demonstration of intrathecal-specific antibody synthesis^c^	Detection of *B. burgdorferi s.l.* by culture and/or PCR from CSF. Intrathecal synthesis of total IgM and/or IgG and/or IgA. Specific serum antibodies. Recent or concomitant EM.
LA	Recurrent attacks or persisting objective joint swelling in one or a few large joints. Alternative explanations must be excluded.	Specific serum IgG antibodies, usually in high concentrations	Synovial fluid analysis. Detection of *B. burgdorferi s.l.* by PCR and/or culture from synovial fluid and/or tissue.
LC	Acute onset of atrioventricular (I–III) conduction disturbances, rhythm disturbances, and sometimes myocarditis or pancarditis. Alternative explanations must be excluded.	Specific serum antibodies	Detection of *B. burgdorferi s.l.* by culture and/or PCR from endomyocardial biopsy. Recent or concomitant EM and/or neurologic disorders.
Ocular manifestations	Conjunctivitis, uveitis, papillitis, episcleritis, keratitis.	Specific serum antibodies	Recent or concomitant LB manifestations. Detection of *B. burgdorferi s.l.* by culture and/or PCR from ocular fluid.

European Commission Definition for LNB (European Commission, 2018)			
Term	Clinical criteria	Laboratory criteria	
LNB	Early LNB: Neurologic symptoms for <6 months With manifestations confined to the PNS (cranial nerves, spinal roots, or peripheral nerves) (Bannwarth syndrome) With CNS manifestations Late LNB: neurologic symptoms for >6 months With PNS manifestations With CNS manifestations	Pleocytosis in CSF AND evidence of intrathecal production of LB antibodies, OR *B burdgorferi s.l.* isolation, OR nucleic acid detection in CSF, OR detection of IgG LB antibodies in blood specimen only for children (<18 years) with facial palsy or other cranial neuritis and a recent (<2 months) history of EM	

The U.S. Centers for Disease Control and Prevention case definitions (Centers for Disease Control and Prevention, 1997)			
Clinical description	A systemic, tick-borne disease with protean manifestations, including dermatologic, rheumatologic, neurologic, and cardiac abnormalities. The best clinical marker for the disease is the initial skin lesion (*i.e.*, EM) that occurs in 60–80% of patients.		
Laboratory criteria for diagnosis	Isolation of *B. burgdorferi* from a clinical specimen or demonstration of diagnostic IgM or IgG antibodies to *B. burgdorferi* in serum or CSF. A two-test approach using a sensitive enzyme immunoassay or immunofluorescence antibody followed by Western blot is recommended.		
Case classification—confirmed	(a) A case with EM or (b) a case with at least one late manifestation that is laboratory confirmed.		
EM	For purposes of surveillance, EM is defined as a skin lesion that typically begins as a red macule or papule and expands over a period of days to weeks to form a large round lesion, often with partial central clearing. A single primary lesion must reach ≥5 cm in size. Secondary lesions also may occur. Annular erythematous lesions occurring within several hours of a tick bite represent hypersensitivity reactions and do not qualify as EM. For most patients, the expanding EM lesion is accompanied by other acute symptoms, particularly fatigue, fever, headache, mildly stiff neck, arthralgia, or myalgia. These symptoms are typically intermittent. The diagnosis of EM must be made by a physician. Laboratory confirmation is recommended for persons with no known exposure.		
Late manifestations	Late manifestations include any of the following when an alternate explanation is not found. (1) Musculoskeletal system. Recurrent, brief attacks (weeks or months) of objective joint swelling in one or a few joints, sometimes followed by chronic arthritis in one or a few joints. Manifestations not considered as criteria for diagnosis include chronic progressive arthritis not preceded by brief attacks and chronic symmetrical polyarthritis. In addition, arthralgia, myalgia, or fibromyalgia syndromes alone are not criteria for musculoskeletal involvement. (2) Nervous system. Any of the following, alone or in combination: lymphocytic meningitis; cranial neuritis, particularly facial palsy (may be bilateral); radiculoneuropathy; or, rarely, encephalomyelitis. Encephalomyelitis must be confirmed by demonstration of antibody production against *B. burgdorferi* in the CSF, evidenced by a higher titer of antibody in CSF than in serum. Headache, fatigue, paresthesia, or mildly stiff neck alone are not criteria for neurologic involvement. (3) Cardiovascular system. Acute onset of high-grade (2° or 3°) atrioventricular conduction defects that resolve in days to weeks and are sometimes associated with myocarditis. Palpitations, bradycardia, bundle branch block, or myocarditis alone are not criteria for cardiovascular involvement.		

^a^ If <5 cm in diameter, a history of tick bite, a delay in appearance (after the tick bite) of at least 2 days, and an expanding rash at the site of the tick bite is required.

^b^ As a rule, initial and follow-up samples must be tested in parallel to avoid changes by interassay variation.

^c^ In early cases, intrathecally produced specific antibodies may still be absent.

CNS, central nervous system; CSF; cerebrospinal fluid; EM, erythema migrans; EUCALB, European Concerted Action on Lyme Borreliosis; IgA, immunoglobulin A; IgG, immunoglobulin G; IgM, immunoglobulin M; LA, Lyme arthritis; LB, Lyme borreliosis; LC, Lyme carditis; LNB, Lyme neuroborreliosis; PCR, polymerase chain reaction; PNS, peripheral nervous system.

Identifying geographic areas where incidence rates of LB disease are highest could inform future intervention strategies. Current reviews have only focused on Western Europe (ECDC, 2018; Sykes and Makiello, 2017), and there is a need to determine if LB is an emerging public health problem in the rest of Europe as well. To inform intervention strategies in Europe, including vaccines under development, we systematically assessed publicly available surveillance data for LB incidence in all of Europe.

## Methods

We obtained publicly available surveillance data that report the incidence of LB in all regions of Europe (PROSPERO CRD42021236906) to develop country-level estimates of the incidence of LB in Europe over the past 15 years. Websites from the following sources were searched for public health surveillance reports on LB from 2005 to 2020: ECDC (ECDC, 2020), World Health Organization (World Health Organization, 2000), and other websites of government public health agencies and institutes in Europe with LB surveillance programs and annual reports (Supplementary Table S1). No limit was placed on language; non-English reports were translated from their original published language into English using *DeepL Translator Pro 2021*. Data for Germany were not published as reports, but were obtained from regional surveillance data collected centrally by the Robert Koch Institute and available on-line (Robert Koch Institute, 2021a).

### Analysis

Data were extracted into Excel. Population-based incidence was the number of reported LB cases per 100,000 population per year (PPY). When public health surveillance reports only provided the number of cases of LB, we transformed the number of cases into incidence proportions greed (Dicker et al, 2012) using available census data for European populations. For the purposes of analysis, we considered Europe as comprising four major regions: Eastern Europe, Northern Europe (subdivided as the Baltic states, Nordic countries, and The United Kingdom and Ireland), Southern Europe, and Western Europe. Country-level data were then organized accordingly (Table 2) (World Health Organization, 2022).

**Table 2.** Results of Search for Publicly Available, National-Level Surveillance Providing Estimates of Lyme Borreliosis Incidence

Region	No. of countries extracted	No. of countries in Europe by region^a^	Countries extracted	Countries with NO surveillance information extracted
Eastern Europe	8	13	Bulgaria, Czech Republic, Hungary, Poland, Romania, Russia, Slovakia, Slovenia	Azerbaijan, Belarus, Georgia, Republic of Moldova, Ukraine
Northern Europe				
Baltic states	3	3	Estonia, Latvia, Lithuania	*—*
Nordic Region	2	5	Finland, Norway	Denmark, Iceland, Sweden
United Kingdom and Ireland	5	5	England, Scotland, North Ireland, Ireland, Wales	*—*
Southern Europe	3	17	Croatia, Portugal, Serbia	Albania, Andorra, Armenia, Bosnia and Herzegovina, Cyprus, Italy, Greece, Kosovo, Malta, Montenegro, San Marino, Spain, Republic of Macedonia, Turkey
Western Europe	4	9	Belgium, France, Germany, Switzerland	Austria, Liechtenstein, Luxembourg, Monaco, Netherlands
Total countries	25	52		

^a^ There were 25 of 52 countries (48.1%) with publicly available, national-level surveillance data providing estimates of LB incidence.

**FIG. 1.**
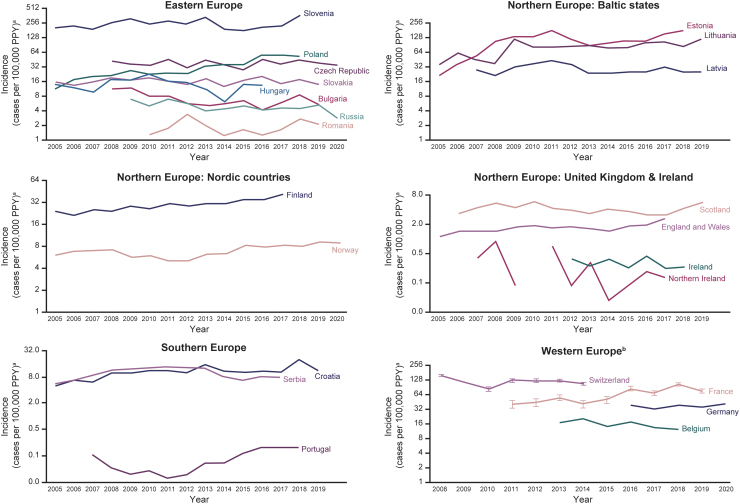
Incidence^a^ of LB from national surveillance networks (2005–2019). ^a^Number of LB cases per 100,000 PPY, shown in logarithmic scale base 2 for comparison. ^b^From sentinel surveillance data. Vertical lines show 95% CIs for extrapolated data. Gaps in data occur when no data reported for year(s). LB case definitions changed in Scotland in 2012**–**2013 (to not require laboratory confirmation) and in Poland in 2005 (to reflect EUCALB and U.S. CDC case definitions). Switzerland stopped reporting LB surveillance data in 2014. The United Kingdom and Ireland data are derived *ad hoc* from routine surveillance data from each of the individual constituent countries. Tabulated data are provided in Supplementary Table S2. CDC, U.S. Centers for Disease Control and Prevention; CI, confidence interval; EUCALB, European Concerted Action on Lyme Borreliosis; LB, Lyme borreliosis; PPY, population per year.

We plotted trends of incidence estimates by country, grouped by European region, over time (2005–2019) for all years of available data. Graphs were log transformed in logarithmic scale base 2 for comparison. When incidence estimates were extrapolated from sentinel surveillance systems, we presented the confidence intervals (95% CIs) around these estimates. The 95% CI for mean incidence was calculated based on the *F*-distribution method (Waller et al, 1994) (Fig. 1 and Supplementary Table S2).

**FIG. 2.**
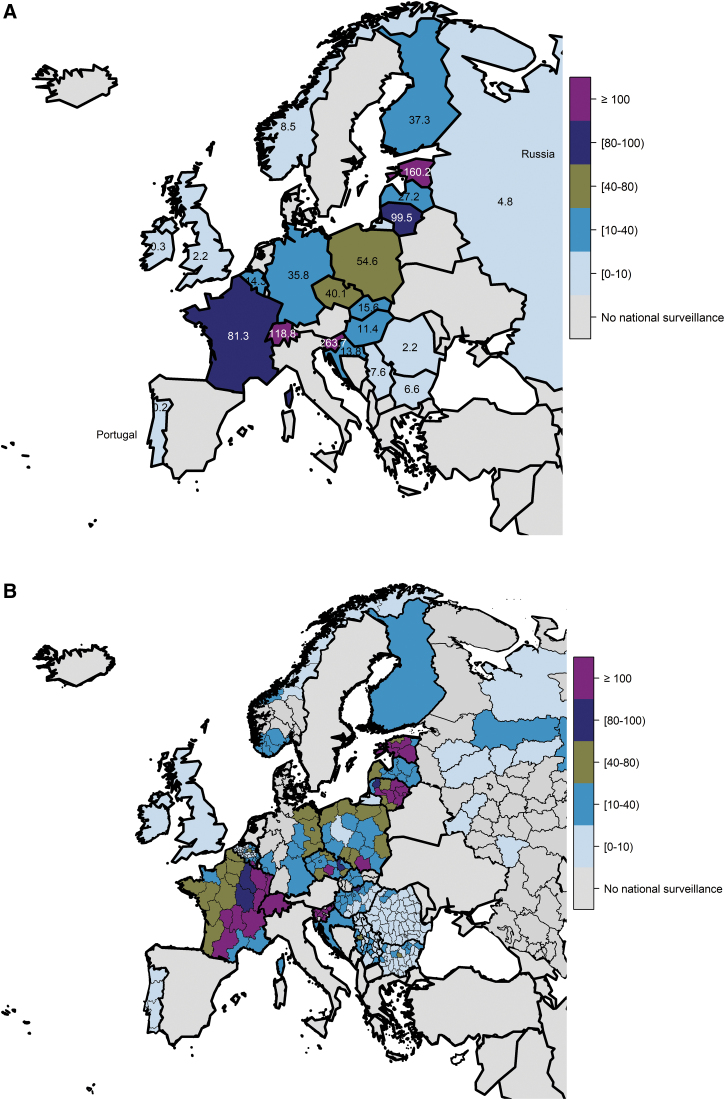
Incidence^a^ (cases per 100,000 PPY) of LB overall by country from national surveillance networks in Europe **(A)** for the national average and **(B)** for the subnational level (when data available). *Maps represent national surveillance data available for European countries, based on our search methodology. ^a^Weighted mean for most recent 3-year period available (all case definitions).

We mapped incidence using the mean of the most recent 3 years of available incidence data from surveillance weighted by annual population. Population-based, national incidence estimates were mapped overall for all European countries (Fig. 2A). Subnational incidence estimates were also mapped from available data identified among European countries (Fig. 2B) and organized by European regions (Supplementary Figs. S1–S4).

We also estimated the total population in Europe that resided in a geographic area defined as high LB risk. As we were unable to identify a definition of high LB incidence from the ECDC or other publicly available sources, we used the United States Centers for Disease Control and Prevention (CDC) threshold (LB incidence >10 cases per 100,000 PPY) (Centers for Disease Control and Prevention 2022a; Centers for Disease Control and Prevention 2022b; Centers for Disease Control and Prevention 2021a; Schwartz et al, 2017).

We summed all national and subnational data from countries with surveillance data indicating a consistent incidence of >10 per 100,000 PPY (Supplementary Table S3). The total European population was obtained by summing the 2020 Eurostat census data for all 52 European countries, or from the most recent year before 2020 if 2020 census data were not available for that country (Eurostat, 2022). We divided the population living in areas with high incidence of LB by the total European population, multiplied by 100, to obtain the percentage of populations at risk for LB in Europe. Analyses were performed using the statistical software R; we used the same tool to develop maps (R Core Team, 2016).

## Results

National-level public health surveillance estimates of LB incidence were available for 25 of 52 (48.1%) European countries (Table 2). This included 8/13 (61.5%) countries in Eastern Europe, 10/13 (77%) in Northern Europe, 3/17 (17.6%) in Southern Europe, and 4/9 (44.4%) in Western Europe. In the Northern Europe subregions, we obtained information from 3/3 (100%) countries in the Baltic states, 2/5 (40%) in Nordic countries, and 5/5 (100%) in the United Kingdom and Ireland. Incidence trends from surveillance data are provided (Fig. 1), organized by European region. Maps of overall incidence by country are presented at the national level (Fig. 2A) and at the subnational level (Fig. 2B). Subnational incidence by European region is presented in maps (Supplementary Figs. S1–S4). Approximately 128,888 cases are reported annually from the countries that have surveillance systems.

### Case definitions in European countries

Definitions used across European countries were highly variable and included cases of LC, consultations for tick bite or EM, LNB confirmed by analysis of cerebrospinal fluid, LB as coded by International Classification of Diseases (ICD) or Read codes (codes associated with clinical terms of disease developed by the National Health System [NHS] England) (NHS Digital, 2022), laboratory-confirmed cases, or clinical cases. Only two countries (France and Poland) employed EUCALB definitions in their national routine surveillance systems (Stanek et al, 2011). The case definition for LNB, reportable to the ECDC since 2018 (European Commission, 2018), was used by two countries, Bulgaria and Romania. One country (Poland) used both the U.S. CDC *and* EUCALB case definitions (Tables 1 and 3).

**Table 3.** Summary of National Surveillance Systems in European Countries Reporting Lyme Borreliosis Incidence Data Between 2005 and 2020

Country	Surveillance type	Case definition	Case definition classifications
Eastern Europe			
Bulgaria	Passive: clinician reported	LB Clinical criteria: EM, development of regional lymphangitis and lymphadenitis, and subsequent staged involvement of the following organs and systems: Musculoskeletal system: recurrent episodes of swelling and pain in at least one of the large joints Heart: acute arrhythmias (AV block I–III degree), rarely myocarditis and pancarditis Eyes: conjunctivitis, uveitis, papillitis, episcleritis, keratitis Laboratory criteria: specific antibodies in serum, nucleic acid detection, isolation of *B. burgdorferi* from clinical material LNB Clinical criteria: neurologic symptoms according to the proposed EFNS definition Laboratory criteria: confirmed case of pleocytosis in the CSF AND evidence of intrathecal antibody production, OR isolation of the bacterium or its DNA in the CSF, OR only for children (younger than 18 years) with facial paralysis or other cranial neuritis and a recent (<2 months ago) manifestation of EM; detection antibodies in serum Probable case: CSF pleocytosis and positive CSF serology OR specific intrathecal antibody production	Clinically diagnosed and/or laboratory-confirmed LB cases Laboratory-confirmed LNB cases using European Commission case definition
Czech Republic	Passive: clinician reported	Clinical definition: Early localized: EM Early disseminated: causative agent in skin tissue (lymphocytoma), musculoskeletal, nerve, and cardiac Late chronic: months to years after infection with nervous system (chronic encephalomyelopathy, chronic polyneuritis, depression, and other psychiatric manifestations), joint (LA), and skin (inflammatory or ACA) involvement Laboratory criteria: IgM and IgG antibodies in serum, CSF, synovial fluid by ELISA with clinically controversial cases confirmed by Western blot; cultivation of the bacterium from clinical material; detection of *Borrelia* antigens or detection of genomic and plasmid DNA, possibly in combination with direct microscopic detection Epidemiologic criteria: at least one of the following within 4 weeks before symptom onset: confirmed tick bite, stayed in a tick area, risk of handling a tick, or removal of a tick when there is direct contact with the patient's skin	Clinically diagnosed and/or laboratory-confirmed LB cases
Hungary	Passive: clinician reported	Probable case: EM or clinical criteria for early LNB when epidemiologic conditions are met Confirmed case: at least one clinical and one laboratory condition are met Clinical conditions: EM Early LNB: one of the following: acute radiculoneuritis, acute paralysis of a brain nerve, or meningitis Epidemiologic conditions: tick bite during incubation period Laboratory conditions: EM: no laboratory test required Early LNB: intrathecal antibodies and/or isolation of *B. burgdorferi* or detection of DNA in a clinical specimen	Clinically diagnosed and/or laboratory-confirmed LB cases
Poland	Passive: clinician and laboratory reported	LNB Clinical criteria: neurologic symptoms or diseases unrelated to other obvious causes, most commonly lymphocytic meningitis, encephalomyelitis, cranial neuritis, acute root pain, polyneuritis and polyarthritis, plexitis, or paresis Laboratory criteria Confirmed case: pleocytosis in CSF AND at least one of intrathecal antibodies OR detection of the bacterium or its DNA in CSF, OR only for children <18 years with facial palsy or other cranial neuritis and a recent (<2 months) history of EM; IgG in serum Probable case: pleocytosis in CSF AND detection of antibodies in CSF OR intrathecal antibody production in CSF LB Clinical criteria: Any person with one or more symptoms of EM Osteoarticular system: recurrent episodes of large joint inflammation with swelling, rarely progressing to chronic arthritis Circulatory system: grade I to III atrioventricular block, cardiac arrhythmias, myocarditis, or pericarditis Ocular manifestations: conjunctivitis, uveitis, intraocular optic neuritis, scleritis, or keratitis Lymphocytoma Atrophic dermatitis Laboratory criteria: Confirmed case: LB of the osteoarticular system OR atrophic dermatitis: at least one of positive serology (standard two-tier method) or isolation of the bacterium or its DNA in clinical specimens LD of the circulatory system, lymphocytoma, OR ocular manifestations: at least one of significant increase in specific IgM or IgG antibodies (standard two-tier method) or isolation of the bacterium or its DNA in clinical specimens	Clinically diagnosed and/or laboratory-confirmed LB cases using U.S. CDC and EUCALB case definition
Romania	Passive: clinician and hospital reported	Other than LNB Possible: meets clinical criteria Probable stage 1 disease only: meets the clinical AND epidemiologic criteria (tick bite with 2–30 days before the onset of EM and/or systemic manifestations) Confirmed: meets the clinical AND laboratory criteria (positive culture or serology for *B. burgdorferi*) LNB Probable: meets clinical criteria (EM or symptoms of disseminated disease) and at least one laboratory criterion (pleocytosis in CSF AND detection of IgM or IgG antibodies in serum and/or CSF without demonstration of intrathecal antibodies) Confirmed: meets clinical criteria (EM or symptoms of disseminated disease) and at least one laboratory criteria (pleocytosis in CSF AND evidence of intrathecal production of antibodies OR isolation of the bacterium or its DNA in CSF OR in children <18 years with facial paralysis or other cranial neuritis and recent history (<2 months) of EM—detection of serum IgG antibodies	Laboratory-confirmed LB cases Laboratory-confirmed LNB cases using European Commission case definition
Russia	Passive: clinician reported	LD diagnosis is based on history of tick exposure or exposure to an endemic area; EM, clinical picture, and dynamics development Final diagnosis must be laboratory confirmed by isolation of the bacterium, PCR detection of DNA in blood or CSF, or IgM and IgG in sera (ELISA) confirmed by paired sera	Clinically diagnosed and/or laboratory-confirmed LB cases
Slovakia	Passive: clinician reported	ICD-10 codes: (*B. burgdorferi s.l.*) A69.2, G01, G63.0, M01.2 The disease is confirmed based on the clinical picture and by positive standard two-tier testing	Clinically diagnosed and laboratory-confirmed LB cases
Slovenia	Passive: clinician and laboratory reported	ICD-10 codes: (*B. burgdorferi s.l.*) A69.2, G01, G63.0, M01.2 Reporting: report a probable case (EM only) and a confirmed case (all other clinical forms of LD) Probable case: EM Confirmed case: EM, lymphocytoma, ACA, other skin lesions, LNB, LA, LC, ocular changes, and laboratory criteria: EM: isolation of bacteria or DNA from a skin biopsy Lymphocytoma: evidence of specific antibodies, skin histology consistent with lymphocytoma isolation of bacteria or DNA from a skin biopsy ACA: high titer of specific IgG antibodies, skin histology consistent with ACA, isolation of bacteria or DNA from a skin biopsy Other skin changes: isolation of bacteria or DNA from a skin biopsy LNB: CSF pleocytosis and intrathecal production of specific antibodies, isolation of bacteria or its DNA from CSF, intrathecal production of specific IgM and/or IgG and/or IgA, evidence of specific antibodies in serum LA: detection of specific IgG antibodies in serum, isolation of the bacterium or its DNA from synovial fluid and/or tissue LC: detection of specific IgG antibodies in serum, isolation of the bacterium or its DNA from an endomyocardial biopsy Eye changes: detection of specific IgG antibodies in serum, isolation of the bacterium or its DNA from ventricular fluid	Clinically diagnosed and laboratory-confirmed LB cases
Northern Europe			
Baltic states			
Estonia	Passive: clinician reported	ICD-10 code: A69.2 Lyme disease then laboratory confirmed	Clinically diagnosed and laboratory-confirmed LB cases
Latvia	Passive: clinician reported	ICD-10 codes: (*B. burgdorferi s.l.*) A69.2, G01, G63.0, M01.2	Clinically diagnosed LB cases
Lithuania	Passive: clinician reported	ICD-10 code: A69.2 Lyme disease (EM) The diagnosis is based on clinical symptoms and laboratory tests	Clinically diagnosed and/or laboratory-confirmed LB cases
Nordic countries			
Finland	Passive: clinician and laboratory reported	ICD-10 code: A69.2 No laboratory testing required for clinical EM Microbiologically confirmed LB**:** specific IgG and/or IgM antibodies in serum and/or CSF (two-tier system)	Clinically diagnosed and/or laboratory-confirmed LB cases
Norway	Passive: clinician reported Localized EM not notifiable	Criteria for notification are a clinically compatible case (not just EM) with laboratory detection of the bacterium or its DNA or antibody (IgM or IgG) in CSF or in serum EM is not notifiable; however, multiple EM is considered disseminated disease and must be reported	Laboratory-confirmed LB cases
United Kingdom and Ireland			
England and Wales	Passive: laboratory reported	EM: no laboratory testing required LB without EM: positive by two-tier testing	Clinically diagnosed and/or laboratory-confirmed LB cases
Northern Ireland			
Scotland			
Ireland	Passive: clinician and hospital reported (LNB)	LNB Probable case: meets clinical criteria and at least one laboratory criterion for probable cases Confirmed case: meets clinical criteria and at least one laboratory criterion for confirmed cases Clinical criteria: Neurologic symptoms according to EFNS-suggested case definition Laboratory criteria: Probable case: pleocytosis in CSF AND positive CSF serology OR specific intrathecal antibody production Confirmed case: pleocytosis in CSF AND intrathecal antibody production, OR isolation of the bacterium or its DNA in CSF, OR only for children <18 years with facial palsy or other cranial neuritis and a recent (<2 months) history of EM; IgG in serum	Laboratory-confirmed LNB cases using European Commission case definition
Southern Europe			
Croatia	Passive: clinician reported	Possible case: EM Probable case: EM and a history of tick bites or another clinical manifestation (except neurologic) with a history of EM Confirmed case: one of the clinical manifestations (except neurologic) that is also laboratory confirmed by either specific IgM and IgG antibodies in serum or CSF by IFA, EIA, WB; isolation of the bacterium or its DNA	Clinically diagnosed and/or laboratory-confirmed LB cases
Portugal	Passive: clinician reported	Clinical criteria: EM and fever; general malaise, fatigue, headaches, stiffness of the back of the neck, myalgias, migratory arthralgias, lymphadenopathy Late manifestations**:** polyarthritis with a preference for the large joints, chronic arthritis, aseptic meningitis, cranial neuritis, encephalomyelitis, meningoencephalitis, radiculopathies (radiculoneuropathy), auriculoventricular block, myocarditis Laboratory criteria: Isolation of the bacterium or its DNA from a biological sample Positive serology (two-tier method) in serum or CSF Epidemiologic criteria: Confirmed tick bite within 32 days before onset of first symptoms Epidemiologic link to animals with confirmed infection (residence or visit in areas where LB circulates in rodents or deer)	Clinically diagnosed and/or laboratory-confirmed LB cases
Serbia^a^	Passive: network of public health institutes	No established case definition. ICD-10 codes: A69.2 Lyme disease (EM)	Clinically diagnosed and/or laboratory-confirmed LB cases
Western Europe			
Belgium^b^	Sentinel: GPs and laboratories	Laboratory case definition: only if both serologic screening and immunoblot are positive	Laboratory-confirmed LB cases
France	Sentinel: GP reported	Clinical diagnosis: EM Clinical diagnosis with serologic confirmation: presence of neurologic, joint, cutaneous, or cardiac manifestations suggestive of LB in a patient with positive serology (standard two tier)	Clinically diagnosed and/or laboratory-confirmed LB cases (using EUCALB case definitions)
Germany^c^	Passive: clinician and laboratory reported	Any of the three following manifestations: clinically diagnosed EM (ICD-10 code, A69.2) OR laboratory-confirmed LA (ICD-10 code, M01.2) OR laboratory-confirmed acute neuroborreliosis (ICD-10 code, G01)	Clinically diagnosed and/or laboratory-confirmed LB cases
Switzerland	Sentinel: GP reported	No mandatory notification; no established case definition; seasonal situation reports provide information on acute cases; stopped reporting in 2014	Clinically diagnosed and/or laboratory-confirmed LB cases

^a^ There is no national surveillance in Serbia, but data are passively collected in the form of summary reports on a weekly and monthly basis and within the annual reports of 24 institutes of public health in Serbia, which are competent in 25 districts.

^b^ Belgium sentinel laboratory network covers ∼50% of all laboratory tests conducted in Belgium (Bleyenheuft et al, 2015).

^c^ Currently, 9 out of the 16 German federal states covering 42% of the total German population have implemented mandatory notification for EM, LNB, and LA under state-specific regulations.

ACA, acrodermatitis chronica atrophicans; AV, atrioventricular; CDC, U.S. Centers for Disease Control and Prevention; CSF: cerebrospinal fluid; ECDC, definition from the European Centre for Disease Prevention and Control; EFNS: European Federation of Neurological Societies; EIA, enzyme immunoassay; ELISA, enzyme-linked immunosorbent assay; EM, erythema migrans; EUCALB, European Concerted Action on Lyme Borreliosis; GPs, general practices; ICD, International Classification of Diseases; IFA, immunofluorescence assay; IgA, immunoglobulin A; IgG, immunoglobulin G; IgM, immunoglobulin M; LA, Lyme arthritis; LB, Lyme borreliosis; LC, Lyme carditis; LD, Lyme disease; LNB, Lyme neuroborreliosis; WB, Western blot.

All countries have *mandatory* reporting requirements except Belgium, France, Serbia, and Switzerland.

### Incidence of LB

#### Eastern Europe (Bulgaria, Czech Republic, Hungary, Poland, Romania, Russia, Slovakia, and Slovenia)

National surveillance data were available for eight countries in Eastern Europe (Fig. 1). The national incidences of LB in these eight countries considering any case definition ranged from <1 to 365 per 100,000 PPY.

Surveillance data reporting in Eastern Europe includes clinically diagnosed and laboratory-confirmed LB cases (Czech Republic, Hungary, Russia, and Slovenia), EUCALB and the U.S. CDC (Poland), laboratory-confirmed LB cases (Romania, Slovakia), laboratory-confirmed LNB cases using the European Federation of Neurological Societies case definition (Bulgaria), and laboratory-confirmed LNB cases using the ECDC case definition (Romania). Based on surveillance data obtained (25 of 52 European countries), the national incidence of LB was <15 per 100,000 PPY in most years in Bulgaria, Hungary, Romania, Russia, and Slovakia. The incidences were 20–40 per 100,000 PPY in Czech Republic and Poland and >200 per 100,000 PPY in Slovenia, which had a peak incidence of 365 per 100,000 PPY in 2018 (Fig. 2).

Although the incidence of LB was stable from 2005 to 2019 in most countries in Eastern Europe (Hungary, Romania, Russia, Czech Republic, and Slovenia), it increased over time in Bulgaria (until 2012) and Poland (Fig. 2). The reported LB incidences in Poland and Slovakia were lower than in neighboring Czech Republic.

Despite low incidence rates in all countries in Eastern Europe, except Slovenia, there was marked subnational variation in LB incidence in countries in Eastern Europe, with countries with low national LB incidence having areas of high LB incidence. Within-country incidence derived from national surveillance data is provided (Fig. 2 and Supplementary Fig. S1). High incidence (>80 per 100,000 PPY) was observed in all regions of Slovenia, Olomouc and Vysočina in the south and east of the Czech Republic, Nógrád in the north of Hungary, and Malopolskie in the south of Poland.

#### Northern Europe: Baltic states (Estonia, Latvia, and Lithuania)

All three Baltic states (Estonia, Latvia, and Lithuania) conduct public health surveillance for LB recorded as ICD codes (Fig. 2). Surveillance captures laboratory-confirmed LB cases in Estonia, clinically diagnosed and laboratory-confirmed LB cases in Lithuania, and clinically diagnosed LB cases in Latvia.

The incidences of LB in the Baltic states considering any case definition ranged from 21 to 173 per 100,000 PPY. In Latvia, the highest LB incidence was >40 per 100,000 PPY in 2011, compared with >100 per 100,000 PPY in Estonia, and 80–100 per 100,000 PPY in Lithuania. The incidence of LB was stable from 2005 to 2019 across the region (Fig. 2).

Within-country incidence is provided (Fig. 2 and Supplementary Fig. S2). Regions with the highest incidences (>80 per 100,000 PPY) were Saare, Hiiu, Põlva, and Võru in Estonia and Panevezys, Kaunas, and Vlinius in Lithuania. In Latvia, incidence increased from east to west, from 0–20 PPY in the east, to 20–40 per 100,000 PPY in the north, to 40–80 per 100,000 PPY in the western region of Kurzeme.

#### Northern Europe: Nordic countries (Finland and Norway)

Finland and Norway conduct national surveillance of LB (Fig. 2). Clinicians and laboratories report all clinician-diagnosed or laboratory-confirmed LB cases, except for localized EM cases that are not recorded in Norway, even if laboratory confirmed. The reported incidence of LB was 20–40 per 100,000 PPY in Finland and <10 per 100,000 PPY in Norway.

Within-country average incidence rates derived from national surveillance data were available for Norway and are provided (Fig. 2 and Supplementary Fig. S2). National surveillance data suggest marked variation in LB incidence rates at the subnational level, ranging from 0–20 per 100,000 PPY to 30–40 per 100,000 PPY in Agder, located at the southern tip of Norway.

#### Northern Europe: United Kingdom (England, Northern Ireland, Scotland, and Wales) and Republic of Ireland

The four countries in the United Kingdom (England, Northern Ireland, Scotland, and Wales) conduct national LB surveillance for laboratory-confirmed LB cases. Each country undertakes its own separate surveillance (England and Wales combined). We derived *ad hoc* surveillance estimates for the United Kingdom as a whole by combining estimates for the individual countries within the United Kingdom. Ireland conducts national surveillance for laboratory-confirmed LNB cases (Fig. 2).

The reported incidences of LB in the four countries of the United Kingdom ranged from 0 to 6 per 100,000 PPY. The incidence of LB was <2 per 100,000 PPY for most years in England, Wales, and Northern Ireland and between 3 and 6 per 100,000 annually in Scotland. The reported incidence of LNB in Ireland was <0.5 per 100,000 PPY (Fig. 2).

The national LB surveillance data from Ireland and the constituent countries within the United Kingdom are not stratified to provide insight at a subnational level. However, they do suggest that, while the burden of LB is low overall, it does vary by individual country in the region, being lowest in Ireland and Northern Ireland and highest in Scotland (Figs. 1, 2, Supplementary Fig. S2, and Supplementary Table S2).

#### Southern Europe (Croatia, Portugal, and Serbia)

Croatia and Portugal conduct national LB surveillance (Fig. 2) that captures clinician-diagnosed cases (including EM) and laboratory-confirmed LB cases. There is no national surveillance in Serbia, but LB diagnoses based on ICD codes are passively collected and published in the annual reports of 24 institutes of public health.

The reported incidences of LB in Croatia, Portugal, and Serbia (considering any case definition) ranged from 0 to 20 per 100,000 PPY. Based on national surveillance data, the incidences of LB were ∼10 per 100,000 PPY in Croatia, <1 per 100,000 PPY in Portugal, and 6–13 per 100,000 PPY in Serbia across most surveillance years (Fig. 2).

#### Western Europe (Belgium, France, Germany, and Switzerland)

Public health surveillance for LB is conducted in Belgium, France, Germany, and Switzerland (Fig. 2). LB surveillance in Belgium, France, and Switzerland is based on a network of sentinel GPs and laboratories who voluntarily report to the National Reference Center (Supplementary Table S1). LB surveillance in Germany is only conducted in 9 of the 16 federal states (Robert Koch Institute, 2021b). The LB surveillance systems in Belgium and Switzerland only include laboratory-confirmed LB cases. The LB surveillance systems in France and Germany include both clinician-diagnosed and laboratory-confirmed LB cases. France uses EUCALB case definitions (Réseau Sentinelles, 2021; Tessier et al, 2018).

The reported incidences of LB across these four countries in Western Europe ranged from 10 to 156 per 100,000 PPY. Across most surveillance years, the incidences of LB were <15 per 100,000 PPY in Belgium, 20–40 per 100,000 PPY in the reporting states in Germany, 40–80 per 100,000 PPY in France, and >100 per 100,000 PPY in Switzerland (Fig. 2).

Surveillance data from Belgium, Germany, and France showed wide subnational variability in LB incidence (Fig. 2 and Supplementary Fig. S4). In Belgium, the highest incidences (40 to >100 per 100,000 PPY) were observed in central (Leuven) and south/southeast locales (Neufchậteau, Virton, and Arlon). In Germany, the incidences of LB ranged from 20–40 per 100,000 PPY to 40–80 per 100,000 PPY in the western states of Mecklenburg-Western Pomerania, Brandenburg, and Saxony. In France, the highest incidences (80 to >100 per 100,000 PPY) were in the south/south-central (Limousin, Midi-Pyrénées, and Auvergne) and eastern regions (Champagne-Ardenne, Lorraine, Alsace, Franche-Comté, and Rhône-Alpes). Subnational data for Switzerland were not available.

### Population living in high-incidence regions

When we applied the CDC definition for high LB incidence (annual incidence of LB >10 cases per 100,000 PPY) (Centers for Disease Control and Prevention 2022a; Centers for Disease Control and Prevention 2022b; Centers for Disease Control and Prevention 2021a; Schwartz et al, 2017), the total population living in these areas across the 25 countries was 202,418,763 persons. The total population living in Europe, based on available census data was calculated as 844,328,483 persons (Eurostat 2022). Thus, an estimated 202 million persons of 844 million inhabitants (24% of European populations in our study) live in a high LB incidence region. When limited to countries with surveillance data only, an estimated 202 million persons of 468,887,790 inhabitants (43.2%) live in a high LB incidence region.

## Discussion

This article provides a comprehensive review of estimates of population-based LB incidence derived from national surveillance systems from 25 (of 52) countries in Europe. Clusters of countries/subnational regions with high LB incidence (>100 per 100,000 PPY) are evident, for example, in Eastern, Northern (Baltic states and Nordic countries), and Western Europe (Fig. 2). However, we observed marked subnational variation in the incidence of LB that was apparent in Bulgaria, Romania, Finland, Ireland, Scotland, Belgium, France, Germany, Poland, and Switzerland.

The absence of surveillance of data in some neighboring countries (*i.e.*, Italy in the south, Belarus adjacent to the Baltic states) leaves gaps in our understanding of the geographic extent of endemic areas. Differences in LB case definitions, data collection methods, and reporting requirements limit the comparability of LB incidence between countries. Estimation of the total number of LB cases in Europe each year or of a Europe-wide incidence rate is not feasible because of the inherent heterogeneity subnationally in the LB burden and the wide array of different surveillance systems and case definitions. With these limitations in mind, the highest national LB incidences (>100 per 100,000 PPY) from 2005 to 2019 were observed in Estonia, Lithuania, and Slovenia. Although several countries, such as Belgium, Czech Republic, France, and Poland, have relatively moderate national incidence values, subnational localities in each country show high incidences (>100 per 100,000 PPY).

EM, which is usually diagnosed clinically without a laboratory confirmation, is a much more common manifestation of LB, while disseminated LB (such as LNB, LC, or LA) diagnosis is confirmed by laboratory confirmation. Therefore, within a specific geographic area, a public health surveillance system that depends on reports of clinician-diagnosed LB cases will likely report a higher LB incidence than a surveillance system that includes only reports of laboratory-confirmed LB cases. Due to the different surveillance systems and varying case definitions used by the European countries included in our study, there are limitations comparing incidence estimates between countries and European regions. Variations in reported disease burden could also be due to differences in surveillance systems, underreporting or overreporting and the case definitions used, disease awareness, and laboratory testing practices. For instance, in the Baltic states, a lower incidence was observed in Latvia than Estonia and Lithuania, despite apparently similar surveillance strategies. Hence, standardization of surveillance systems is urgently needed.

The LB incidences in Poland and Slovakia were lower than in neighboring Czech Republic. Variations in reported LB incidence between neighboring countries have been reported in other studies (Stefanoff et al, 2014) using national surveillance data that reported a 9.3-fold difference in LB incidence between the Czech Republic and Poland, even after adjusting for an “epidemiologic gradient” to account for differences in surveillance systems between the countries.

In several ecological regions, LB incidence varied markedly across national borders, highlighting the impact of surveillance approaches on reported disease rates. In Poland, lower LB incidence has been attributed to differences in the management of LB surveillance compared with the Czech Republic. Progressive improvements in Poland's surveillance system over time have translated into increasing incidence rates that are now approaching those of its neighbor (Stefanoff et al, 2014). Use of both the EUCALB and U.S. CDC case definitions in Poland can be problematic, given the differences in *Borrelia* genospecies and disease burden, distribution, and spread of Lyme disease in the United States compared to Europe (Centers for Disease Control and Prevention, 2022a).

A strength of routine surveillance is its ability to capture trends and changes in trends. Public health surveillance data indicate an increase in the incidence of LB from 2005 to 2019 in selected countries of Northern (Baltic states and Nordic countries) and Eastern Europe. On the other hand, LB incidence was stable from 2005 to 2019 in many of the other countries of Eastern Europe, with evidence of decreasing incidence in Romania.

Surveillance data can be used to estimate the population that resides in a geographic area at increased risk for LB. In the United States, areas with an incidence of >10 that reported confirmed LB cases per 100,000 PPY are considered high incidence areas (Centers for Disease Control and Prevention, 2021b). In this respect, we observed marked subnational variation in the incidence of LB in Bulgaria, Romania, Finland, Ireland, Scotland, Belgium, France, Germany, Poland, and Switzerland. Overall, we estimate that approximately 202 million of 844 million inhabitants (24% of European populations in our study) reside in areas of Europe where the incidence is >10 per 100,000 PPY (Eurostat 2022). Specific to countries where surveillance data were collected, approximately 202 million of 469 million inhabitants (43.2%) reside in areas of high LB incidence. Currently, there is no established threshold to identify high LB incidence regions in Europe; thus, the U.S. CDC threshold was applied in this analysis. It is conceivable that this threshold may not be valid for Europe particularly due to lack of consistency in case definitions and reporting systems and to variations in relative prevalence of *Borrelia* genospecies that leads to a different spectrum of illness compared to the United States.

The lack of systematic LB surveillance systems based on standard case definitions is a major impediment to understanding the Europe-wide burden of LB. At a minimum, all countries should work toward nationwide mandatory reporting of all clinician-diagnosed and laboratory-confirmed LB cases. Currently, only 14 (of 52 total) European countries have such surveillance (Nagarajan et al, 2022). Surveillance systems that only include laboratory-confirmed LB cases (or only include LNB cases) fail to capture a substantial proportion of LB cases, which hinders interpretation of regional differences in LB incidence. In addition to differences in surveillance methods, differences in disease awareness among health care providers and differences in laboratory testing approaches also hinder interpretations of data at the Europe-wide level.

The EUCALB published LB case definitions were intended for implementation throughout Europe in 2009 (Stanek et al, 2011); however, their application to date has been limited, as further demonstrated by this review, to France and Poland. The European Commission mandated LNB surveillance by its member states, with reporting to ECDC in 2018 (The Lancet Editorial Board, 2018). LNB is a manifestation of severe LB most likely to be clinically identified and amenable to a standardized case definition (Van den Wijngaard et al, 2017). While LNB reflects a small and variable portion of all LB burden, given that it occurs in only 3–15% of patients with LB, its reflection as a severe clinical manifestation of the disease could translate into potential utility as a standard leading indicator in tracking temporal or local changes in disease burden (Hildenbrand et al, 2009; Koedel et al, 2015; Tuerlinckx and Glupczynski, 2010). This differs between continents due to variability in genospecies of *Borrelia*. In North America, *B. burgdorferi ss* can result in up to 10–20% of patients developing LNB (Eddens et al, 2019; Ford and Tufts, 2021).

Further understanding of the LB disease burden in Europe also would be enhanced by making other clinical manifestations (not just LNB) notifiable to ECDC. Improved EM reporting has the potential to add value to LNB surveillance and serve as a key indicator for LB surveillance; early clinical identification may also help prevent late manifestations and disseminated stages of LB (Blanchard et al, 2022; Van den Wijngaard et al, 2017).

## Conclusions

Surveillance of LB in Europe is only conducted in 25 out of 52 countries (48.1%) and has been hindered by differences in national standards and reporting conventions. Implementation of standardized case definitions in the surveillance systems in Europe will be important for establishing the true incidence of LB and tracking LB disease burden in Europe in relation to future interventions—including vaccination. To this end, reconsideration of national surveillance strategies, including wider implementation of standardized case definitions (such as those published by EUCALB) to optimize their utility, is urgently needed before the availability of any potential vaccine.

## Supplementary Material

Supplemental data
