# Supplementary material for: Incidence of Lyme Borreliosis in Europe from National Surveillance Systems (2005–2020)
Source: Vector Borne Zoonotic Dis. 2023 Apr 12;23(4):156–71. doi: 10.1089/vbz.2022.0071 (PMC10122223; doi:10.1089/vbz.2022.0071)
Supplement: Supplemental data [file Supp_Data.pdf]

Incidence of Lyme Borreliosis in Europe from National Surveillance Systems (2005–2020)

SUPPLEMENT

Table S1. Public health agencies/organizations and corresponding websites for public surveillance reports on Lyme borreliosis in European countries

Country	Public Health Institute responsible for surveillance reports	Main Website URL (Language)	Website URL for Lyme Borreliosis (LB) Surveillance	Location of case definition
**Belgium**	Sciensano	https://nrchm.wiv-isp.be/nl/default.aspx *(Dutch/French)*	https://nrchm.wiv-isp.be/nl/peillabo/default.aspx	https://www.wiv-isp.be/Epidemio/NRC/epilabo/case_definition.pdf
**Bulgaria**	National Center of Infectious and Parasitic Diseases	https://www.ncipd.org/index.php?lang=en (English, Bulgarian)	https://www.ncipd.org/index.php?option=com_k2&view=item&layout=item&id=84&Itemid=1337&lang=bg	https://lex.bg/bg/laws/ldoc/2135508238
	National Statistical Institute (NSI)	https://nsi.bg/en (English, Bulgarian)	https://nsi.bg/bg/search/node?keys=%D0%97%D0%94%D0%A0%D0%90%D0%92%D0%95%D0%9E%D0%9F%D0%90%D0%97%D0%92%D0%90%D0%9D%D0%95	
**Croatia**	Hrvatski Zavod Za Javno Zdravstvo/Croatian Institute of Public Health	https://www.hzjz.hr/en/ (Croatian, English)	https://www.hzjz.hr/tag/zdravstveno-statisticki-ljetopis/	https://www.hzjz.hr/wp-content/uploads/2016/10/hr_definicije.pdf
**Czech Republic**	Státní zdravotní ústav (SZU)/The National Institute of Public Health	http://www.szu.cz/ (Czech, English)	http://www.szu.cz/tema/prevence/infekcni-nemoci	http://www.szu.cz/system-epidemiologicke-bdelosti-lymeske-borreliozy https://www.zakonyprolidi.cz/cs/2008-473
**Estonia**	Republic of Estonia: Health Board (Terviseamet)	https://www.terviseamet.ee/en (Estonia, Russian, English)	https://www.terviseamet.ee/et/nakkushaigused-menuu/tervishoiutootajale/nakkushaigustesse-haigestumine#Nakkushaigustesse%20haigestu	https://www.terviseamet.ee/et/nakkushaigused-menuu/tervishoiutootajale/teatiste-vormid https://www.riigiteataja.ee/akt/110042020002?LeiaKehtiv

**Finland**	Finnish Institute of Health and Welfare (THL)	https://thl.fi/en/web/thlfi-en (Finnish, Swedish, English)	https://thl.fi/fi/web/infektiotaudit-ja-rokotukset/seurantajarjestelmat-ja-rekisterit/tartuntatautirekisteri/tartuntatautien-esiintyvyystilastot/tartuntatautien-esiintyvyys-suomessa-raportit<br><br>https://sampo.thl.fi/pivot/prod/en/infestat/borre/fact_infestat_borre[1]	https://wwwnc.cdc.gov/eid/article/23/8/16-1273_article https://thl.fi/fi/web/infektiotaudit-ja-rokotukset/taudit-ja-torjunta/taudit-ja-taudinaiheuttajat-a-o/borrelia/borrelioosin-seuranta-ja-esiintyvyys-suomessa
**France**	Sentinel Network	http://www.sentiweb.fr/ (French)	https://www.sentiweb.fr/?page=bilan	https://www.sentiweb.fr/france/fr/?page=maladies&mal=18
**Germany**	Robert Koch Institute	https://www.rki.de/EN/Home/homepage_node.html (German, English)	https://www.rki.de/DE/Content/Infekt/Jahrbuch/jahrbuch_node.html[2]	https://www.rki.de/DE/Content/Infekt/EpidBull/Archiv/2009/Ausgaben/05_09.pdf?__blob=publicationFile
**Hungary**	National Center for Epidemiology	http://www.oek.hu/oek.web (Hungarian)	http://www.oek.hu/oek.web?nid=509&pid=3&to=,2475,2465&lang=hun	https://net.jogtar.hu/jogszabaly?docid=99800018.nm https://net.jogtar.hu/getpdf?docid=99800018.nm&targetdate=&printTitle=
	The National Public Health Centre	https://www.nnk.gov.hu/ (Hungarian)	https://www.antsz.hu/felso_menu/temaink/jarvany/Fertozo_betegsegek/Fertozo_eves_jelentesk	
**Republic of Ireland**	Health Protection Surveillance Centre	https://www.hpsc.ie/ (English)	https://www.hpsc.ie/a-z/vectorborne/lymedisease/epidemiologicaldata/	https://www.hpsc.ie/a-z/vectorborne/lymedisease/casedefinition/

[1] Borreliosis in the outpatient treatment notification system (AvoHilmo) & Borreliosis in the infectious disease register
[2] LB does not appear in the Infection epidemiological yearbook on the webpage

**Latvia**	Latvian Center for Disease Prevention and Control	https://www.spkc.gov.lv/lv (Latvian)	https://www.spkc.gov.lv/lv/infekcijas-slimibas-statistika-un-petijumi https://www.spkc.gov.lv/lv/latvijas-veselibas-aprupes-statistikas-gadagramata	https://ssk10.spkc.gov.lv/rsc/SSK_3_sejums_20190922.pdf https://ssk10.spkc.gov.lv/ssk/A692 https://likumi.lv/doc.php?id=20667 (No Laboratory detection)
**Lithuania**	National Public Health Center under the Ministry of Health	https://nvsc.lrv.lt/en (Lithuanian, English)	https://nvsc.lrv.lt/lt/uzkreciamuju-ligu-valdymas/statistika-apzvalgos-1/sergamumo-uzkreciamosiomis-ligomis-apzvalgos	https://e-seimas.lrs.lt/portal/legalAct/lt/TAD/TAIS.204745/asr https://nvsc.lrv.lt/lt/d-u-k/dazniausiai-uzduodami-klausimai-apie-uzkreciamasias-ligas/laimo-liga-1
**Norway**	Norwegian Institute of Public Health (NIPH) Norwegian Surveillance System for Communicable Diseases (MSIS)	https://www.fhi.no/en/ (Norwegian, English) http://www.msis.no/ (Norwegian, English)	https://www.fhi.no/sys/sok/?type=con-32/cat-750,&term=Lyme#main http://www.msis.no/DynamiskRapport.aspx	https://www.fhi.no/contentassets/343f33f920bf4398aff1021acb5e9c18/msis_meldingskriterier_per_09042021.pdf
**Poland**	National Institute of Public Health PZH - National Research Institute	https://www.pzh.gov.pl/ (Polish)	http://wwwold.pzh.gov.pl/oldpage/epimeld/index_p.html#uu<br><br>http://wwwold.pzh.gov.pl/oldpage/epimeld/index_p.html	http://wwwold.pzh.gov.pl/oldpage/epimeld/inne/Def_PL2_6b.pdf
**Portugal**	National Institute of Statistics	https://www.ine.pt/xportal/xmain?xpid=INE&xpgid=ine_main (Portuguese, English)	https://www.ine.pt/xportal/xmain?xpid=INE&xpgid=ine_publicacoes&PUBLICACOEStipo=ea&PUBLICACOEScoleccao=107773&selTab=tab0&xlang=pt	http://www.siis-ram.pt/sinave/Uploads/Anexos/Orientacao_para_autoridade_saude_15_10_20151.pdf
**Romania**	National Institute of Public Health	http://www.cnscbt.ro/ (Romanian)	http://www.cnscbt.ro/index.php/rapoarte-anuale?limit=10&limitstart=0 http://www.cnscbt.ro/index.php/analiza-date-supraveghere/boala-lyme-1?limit=10&limitstart=0 https://insp.gov.ro/rapoarte-si-studii/	https://www.cnscbt.ro/index.php/metodologii/boala-lyme/1394-boala-lyme-metodologie-de-supraveghere-actualizare-11-02-2020/file

**Russia**	Federal Service for Supervision in the field of Consumer Rights Protection and Human Well-Being	https://www.rospotrebnadzor.ru/ (Russian)	https://www.rospotrebnadzor.ru/documents/documents.php?back_url_admin=%2Fbitrix%2Fadmin%2Fiblock_admin.php%3Ftype%3Ddocuments%26lang%3Dru%26admin%3DY&clear_cache=Y&arrFilter_ff%5BNAME%5D=&arrFilter_pf%5BVID_DOC%5D=97&arrFilter_pf%5BNUM_DOC%5D=&arrFilter_pf%5BDAT_DOC%5D=&arrFilter_pf%5BGOD%5D%5BLEFT%5D=&arrFilter_pf%5BGOD%5D%5BRIGHT%5D=&set_filter=Search+&set_filter=Y	https://nnoi.ru/clinical-guid-directed
**Serbia**	Institute of Public Health Serbia's *Dr Milan Jovanovic Batut*	https://www.batut.org.rs/index.php?lang=2 (Serbian, English)	https://www.batut.org.rs/index.php?content=279	https://www.pravno-informacioni-sistem.rs/SlGlasnikPortal/eli/rep/sgrs/skupstina/zakon/2016/15/8/reg
**Slovakia**	Public Health Authority of Slovak Republic	https://www.uvzsr.sk/en/ (Slovak, English)	https://www.uvzsr.sk/index.php?option=com_content&view=category&id=25:vyrona-sprava&layout=blog&Itemid=34&layout=default	https://www.ruvzse.sk/formulare/vs_ruvzse_2017.pdf
**Slovenia**	National Institute of Public Health	https://www.nijz.si/en (Slovene, English)	https://www.nijz.si/sl/nijz/revije/zdravstveni-statisticni-letopis-slovenije	https://www.nijz.si/sl/definicije-prijavljivih-nalezljivih-bolezni-za-namene-epidemioloskega-spremljanja
**Switzerland**	The Sentinel Network	https://www.sentinella.ch/fr/info (French, Italian, English)	http://www.sentinella.ch/fr/publications/<br><br>https://www.bag.admin.ch/bag/de/home/krankheiten/ausbrueche-epidemien-pandemien/aktuelle-ausbrueche-epidemien/zeckenuebertragene-krankheiten.html	https://www.bag.admin.ch/bag/fr/home/krankheiten/infektionskrankheiten-bekaempfen/meldesysteme-infektionskrankheiten/meldepflichtige-ik.html

**England, Wales, Northern Ireland**<br>**Scotland**	Government services and information<br>Health Protection Scotland	https://www.gov.uk/ (English)<br>https://www.hps.scot.nhs.uk/ (English)	https://www.gov.uk/government/publications/zoonoses-uk-annual-reports<br>https://www.hps.scot.nhs.uk/data/latest-epidemiology-reports/	https://www.nice.org.uk/guidance/ng95/resources/lyme-disease-pdf-1837756839877<br><br>https://www.gov.uk/government/collections/lyme-disease-guidance-data-and-analysis<br><br>https://www.gov.uk/government/publications/lyme-borreliosis-epidemiology/lyme-borreliosis-epidemiology-and-surveillance

Table S2. Incidence[a] of lyme borreliosis from national public surveillance reports (2005–2019) by European Region and Country

	Incidence (LB Cases per 100,000 population)														
	**2005**	**2006**	**2007**	**2008**	**2009**	**2010**	**2011**	**2012**	**2013**	**2014**	**2015**	**2016**	**2017**	**2018**	**2019**
**Eastern Europe**															
**Bulgaria**				10.9	11.7	7.9	8.1	5.7	5.2	5.6	6.6	4.1	5.7	8.6	5.4
**Czech Republic**				41.7	36.8	34.2	46.1	31.4	44.2	35.6	27.6	44.4	37.2	44.5	38.5
**Hungary**	14.2	12.2	9.4	18.0	17.3	23.5	16.8	15.3	11.3	6.2	14.4	13.6			
**Poland**	11.5	17.5	20.3	21.7	27.1	23.6	23.8	22.8	33.2	36.0	35.4	55.2	56.0	52.5	
**Romania**						1.3	1.8	3.5	2.1	1.3	1.7	1.3	1.7	2.7	2.1
**Russia**					6.8	5.0	7.0	5.8	4.0	4.4	5.1	4.2	4.6	4.4	5.5
**Slovakia**	15.7	13.6	15.8	19.4	17.1	19.6	15.8	14.0	18.4	12.6	16.8	20.4	14.8	18.0	14.0
**Slovenia**	206.4	222.9	193.0	255.5	308.7	244.1	273.8	239.8	337.0	188.7	181.4	207.0	219.4	364.5	
**Northern Europe**															
Baltic states															
**Estonia**	20.9	35.8	53.7	106.0	133.3	128.4	171.6	115.4	85.7	98.0	106.5	107.9	149.2	173.1	
**Latvia**			27.5	21.5	31.9	37.0	42.1	35.6	22.6	23.5	25.0	24.4	31.5	25.0	25.2
**Lithuania**	34.6	61.7	42.9	35.9	113.1	81.4	79.6	81.2	86.4	76.7	77.1	100.9	99.2	81.6	117.8
Nordic countries															
**Finland**	23.5	21.6	25.0	24.0	28.0	27.0	31.0	29.0	31.0	31.0	35.0	35.0	42.0		
**Norway**	6.0	6.8	7.0	7.3	5.7	5.9	5.0	5.1	6.3	6.3	8.2	7.8	8.3	7.9	9.2
The United Kingdom & Ireland															
**England & Wales**	1.1	1.4	1.5	1.5	1.8	1.9	1.7	1.8	1.6	1.5	1.8	1.9	2.7		
**Ireland**								0.4	0.3	0.4	0.3	0.4	0.3	0.3	
**Northern Ireland**	0	0	0.4	0.9	0.1	0	0.7	0.1	0.3	0.1	0.1	0.2	0.1		
**Scotland**		3.3	4.5	5.5	4.4	5.9	4.3	3.9	3.3	4.2	3.7	3.2	3.1	4.4	5.7
**Southern Europe**															
**Croatia**	5	6.9	6.1	10.1	10	11.4	11.6	10.1	15.5	11.0	10.3	11.1	10.3	19.7	11.5
**Portugal**	0	0	0.1	0.1	0.1	0.1	0	0.1	0.1	0.1	0.1	0.2	0.2	0.2	
**Serbia**	5.6	6.9	8.8	11.7	12.6	13.3	13.7	13.3	13.1	8	6.8	8.4	7.7		
**Western Europe**															
**Belgium**	16.2	13.5	10.1	12.8	11.5	10.4	11	11.7	16.7	20.2	13.9	17.3	13.4	12.2	
**France**					42	42	41	44	55	41	51	84	69	104	76
**Germany**												37.9	32.9	38.8	35.6
**Switzerland**				156	114	83	126	124	123	109					

[a]Incidence proportion is the number of (reported LB confirmed/total) cases per 100,000 population per year.

Note: Gaps indicate no data were reported for that year.

Total population living in Europe, based on available census data (Eurostat Regional Statistics 2022): 844,328,483 (Regional statistics by NUTS classification: Population and area. Eurostat. Updated Jun 02, 2022. Accessed Nov 15, 2022. https://ec.europa.eu/eurostat/databrowser/explore/all/general?lang=en&subtheme=reg.reg_dem.reg_dempoar&display=list&sort=category)

Table S3 Countries and sub-national areas in Europe[a] with average LB incidence >10 per 100,000 PPY and populations estimates of persons at risk

Country	Sub-national area	Average Incidence (per 100,000)	Regional Populations (if >10 per 10,000)	Population at Risk	National Population	% of Population at Risk
Belgium	Flemish Region	12.8	1,068,643.3	1,444,827.7	11,322,022.7	0.1
	Walloon Brabant	41.9	376,184.3			
Bulgaria	Dobrich	14.0	173,928.3	1,099,950.0	7,000,518.3	0.2
	Gabrovo	52.3	108,418.7			
	Lovech	13.0	124,793.3			
	Montana	23.5	129,617.3			
	Pernik	24.3	120,830.3			
	Razgrad	24.6	112,244.0			
	Ruse	14.5	218,456.3			
	Targovishte	14.3	111,661.7			
Czechia	Jihocesky kraj	73.8	1,360,998.0	10,626,430.0	10,653,156.3	1.0
	Jihomoravsky	25.7	550,688.0			
	Karlovarsky	20.7	640,909.0			
	Kraj Vysocina	118.5	1,184,729.0			
	Kralovehradecky	47.6	295,285.0			
	Liberecky	65.9	441,608.0			
	Moravskoslezky	30.5	1,204,346.0			
	Olomoucky	92.8	632,547.0			
	Pardubicky	39.5	519,125.0			
	Plzensky kraj	38.1	582,601.0			
	Prague	17.8	1,301,135.0			
	Stredocesky kraj	31.4	820,580.0			
	Ustecky	28.4	509,019.0			
	Zlin	78.4	582,860.0			
Estonia	Harju	149.6	503,920.0	1,177,920.0	1,179,085.3	1.0
	Hilu	434.4	9,297.7			
	Ida-Viru	19.4	104,906.3			
	Jarva	92.3	30,047.7			
	Jogeva	116.8	29,512.7			
	Laane	334.0	22,348.7			
	Laane-Viru	54.2	56,674.0			
	Parnu	263.7	80,744.7			
	Polva	186.8	26,763.7			
	Rapla	249.3	33,198.7			
	Saare	920.4	33,107.7			
	Tartu	113.4	138,982.7			

	Valga	30.4	27,917.0			
	Vilijandi	175.8	46,604.0			
	Voru	155.5	33,894.7			
Finland	Aland Islands	1980.7	29,750.7	5,451,328.0	5,524,450.0	1.0
	Central Finland	103.1	252,887.0			
	Central Ostrobothnia	45.4	77,763.0			
	Central Savonia	97.0	41,210.3			
	Finland Proper	171.2	481,930.3			
	Helsinki-Uusimaa	119.0	1,671,543.0			
	Kymenlaakson	195.8	166,784.7			
	Lapland	11.1	117,400.7			
	North Karelia	145.1	165,531.0			
	Northern Ostrobothnia	29.2	409,673.7			
	Northern Savonia	98.6	245,655.3			
	Päijät-Hämeen	46.1	211,175.0			
	Pirkanmaa	29.4	535,057.3			
	Satakunta	61.9	218,680.7			
	South Karelia	204.3	128,856.3			
	South Ostrobothnia	21.2	194,600.3			
	Southern Savonia	63.8	100,248.0			
	Tavastia Proper	40.0	171,733.7			
	Vaasa	69.5	169,730.0			
	West Ostrobothnia	13.6	61,117.0			
France	Auvergne-Rhône-Alpes	190.7	8,153,233.0	65,627,454.0	65,627,454.0	1.0
	Bourgogne-Franche-Comté	94.9	2,785,393.0			
	Bretagne	54.3	3,402,932.0			
	Centre-Val de Loire	61.3	2,564,915.0			
	Corse	24.3	349,465.0			
	Grand Est	210.3	5,542,094.0			
	Hauts-De-France	56.2	5,987,172.0			
	Ile-de-France	66.0	12,395,148.0			
	Normandie	32.2	3,307,286.0			
	Nouvelle-Aquitaine	66.3	6,081,985.0			
	Occitanie	104.5	6,053,548.0			
	Pays de la Loire	45.3	3,873,096.0			
	Provence-Alpes-Cote d'Azur	31.3	5,131,187.0			
Germany	Baden-Württemberg	203.0	11,103,043.0	82,509,232.0	83,400,000.0	1.0
	Bavaria (Bayern)	275.0	13,108,586.7			
	Berlin	123.8	3,695,901.7			
	Brandenburg	301.9	2,518,611.0			
	Hamburg	80.3	1,852,478.0			
	Hessen	203.0	6,293,154.0			
	Mecklenburg-Vorpommern	249.4	1,608,627.3			

	Niedersachsen (Lower Saxony)	223.4	8,003,421.0			
	Nordrhein-Westfalen	143.4	17,925,570.0			
	Rheinland-Pfalz	208.5	4,090,807.3			
	Saarland**	33.6	988,129.3			
	Sachsen (Saxony)	447.9	4,074,060.0			
	Sachsen-Anhalt (Saxony-Anhalt)	293.3	2,199,312.7			
	Schleswig-Holstein	146.8	2,910,875.0			
	Thuringen	377.7	2,136,655.0			
Hungary	Csongrad	31.1	406,774.0	2,510,260.8	9,872,900.3	0.3
	Fejer	11.3	417,408.4			
	Komarom-Esztergom	15.5	299,132.2			
	Nograd	84.1	196,056.7			
	Somogy	15.3	312,685.2			
	Vas	21.6	254,383.5			
	Veszprem	23.6	346,189.0			
	Zala	12.4	277,631.8			
Latvia	Kurzeme	49.0	244,896.7	1,942,629.0	1,933,428.6	1.0
	Latgale	16.8	267,692.7			
	Pierigas	33.8	366,953.0			
	Riga	23.0	638,522.0			
	Vidzeme	32.8	190,426.3			
	Zemgale	10.7	234,138.3			
Lithuania	Alytaus	48.2	141,606.7	2,848,454.3	2,828,402.5	1.0
	Kauno	111.4	570,115.0			
	Klaipedos	39.9	320,792.3			
	Marijampoles	48.5	145,241.3			
	Panevezio	129.4	224,920.0			
	Siaullai	75.1	270,759.3			
	Taurages	103.6	98,541.7			
	Telsiai	91.8	137,733.7			
	Utenos	102.7	133,437.7			
	Vilnius	113.4	805,306.7			
Norway	Aust-Agder	32.2	242,401.0	1,714,461.7	5,330,470.3	0.3
	More og Romsdal	24.3	193,574.0			
	Rogaland	12.0	419,968.7			
	Vestfold	15.7	347,342.0			
	Vestland	23.9	511,176.0			
Poland	Greater Poland (Wielkopolskie)	17.3	3,487,578.0	38,426,649.0	38,409,094.0	1.0
	Kuyavian-Pomeranian (Kujawsko-Pomorskie)	23.7	2,096,001.0			
	Lesser Poland (Malopolskie)	99.0	3,395,886.0			
	Lodz	25.1	2,469,948.0			
	Lower Silesian	29.7	2,909,660.0			

	Lublin (Lubelskie)	90.8	2,120,626.0			
	Lubusz (Lubuskie)	61.1	1,023,532.0			
	Masovia (Mazowieckie)	37.4	5,386,169.0			
	Opole (Opolskie)	72.0	986,913.0			
	Podlaskie	115.3	1,181,384.0			
	Pomeranian (Pomorskie)	60.1	2,327,987.0			
	Silesian (Slaskie)	55.1	4,539,820.0			
	Subcarpathian (Podkarpackie)	72.4	2,127,828.0			
	Swietokrzyskie (Holy Cross)	32.3	1,244,310.0			
	Warmian-Masurian (Warminsko-mazurskie)	95.9	1,431,162.0			
	West Pomeranian (Zachodniopomorskie)	49.7	1,697,845.0			
Romania	Maramures	13.1	467,096.3	681,209.7	19,631,264.3	0.0
	Salaj	14.1	214,113.3			
Serbia	Kolubarski	59.6	174,513.0	1,930,283.0	7,093,126.7	0.3
	Mačvanski	30.0	298,931.0			
	Moravički	14.4	212,603.0			
	Pčinjski	11.3	159,081.0			
	Podunavski	17.2	199,395.0			
	Pomoravski	13.5	214,536.0			
	Rasinski	24.8	241,999.0			
	Raški	13.5	309,258.0			
	Zaječarski	12.5	119,967.0			
Slovakia	Banskobystricky	24.6	649,723.7	1,928,134.0	5,442,961.3	0.4
	Trenciansky	24.7	587,354.0			
	Zilinsky kraj	28.1	691,056.3			
Slovenia	Celje	192.3	302,406.0	2,066,594.3	2,067,079.3	1.0
	Koper	231.7	149,326.0			
	Kranj	356.0	203,649.7			
	Ljublijana	236.4	661,481.3			
	Maribor	213.7	321,575.0			
	Mestna Obcina Nova Gorica	467.3	101,264.3			
	Murska Sobota	385.0	115,197.7			
	Novo Mesto	301.5	140,960.0			
	Obcina Ravne na Koroskem	288.3	70,734.3			
**Total**			**221,985,818**			

LB, Lyme borreliosis; PPY, population per year.

[a]For 25 European countries with publicly available surveillance data available 2005–2020.

Figure S1 Sub-national incidence[a] (cases per 100,000 PPY) of LB from national surveillance networks in Eastern Europe

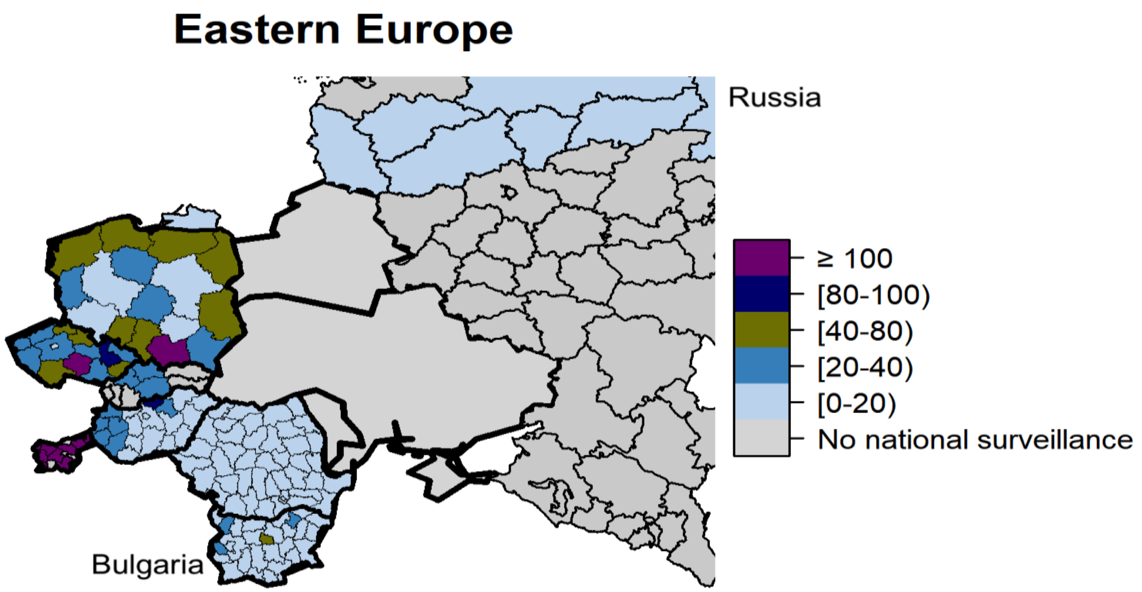

LB, Lyme borreliosis; PPY, population per year.
[a]Weighted mean for the most recent 3-year period available (all case definitions).

Figure S2 Sub-national incidence[a] (cases per 100,000 PPY) of LB from national surveillance networks in Northern Europe

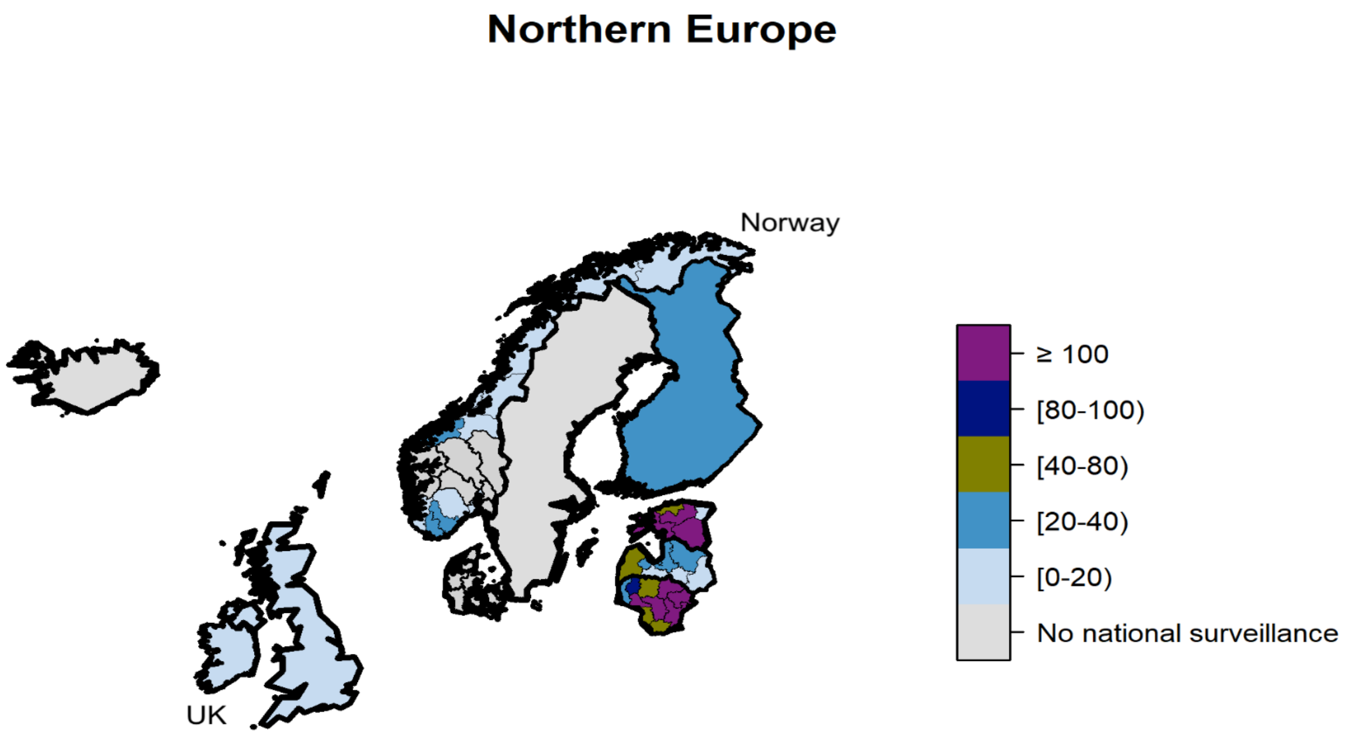

LB, Lyme borreliosis; PPY, population per year.
[a]Weighted mean for the most recent 3-year period available (all case definitions).

Figure S3 Sub-national incidence[a] (cases per 100,000 PPY) of LB from national surveillance networks in Southern Europe

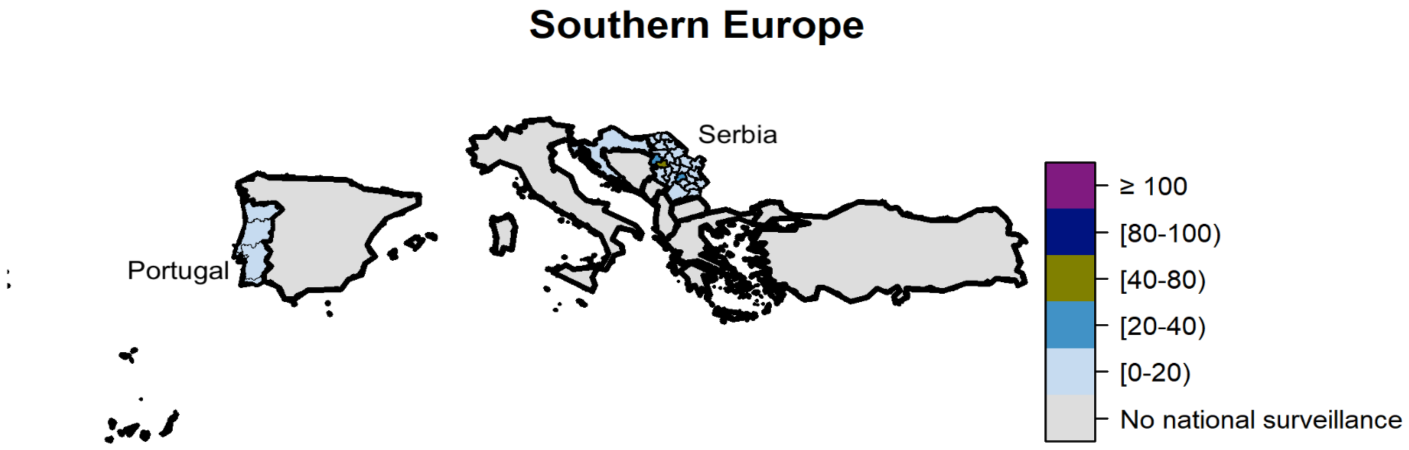

LB, Lyme borreliosis; PPY, population per year.
[a]Weighted mean for the most recent 3-year period available (all case definitions).

Figure S4 Sub-national incidence[a] (cases per 100,000 PPY) of LB from national surveillance networks in Western Europe

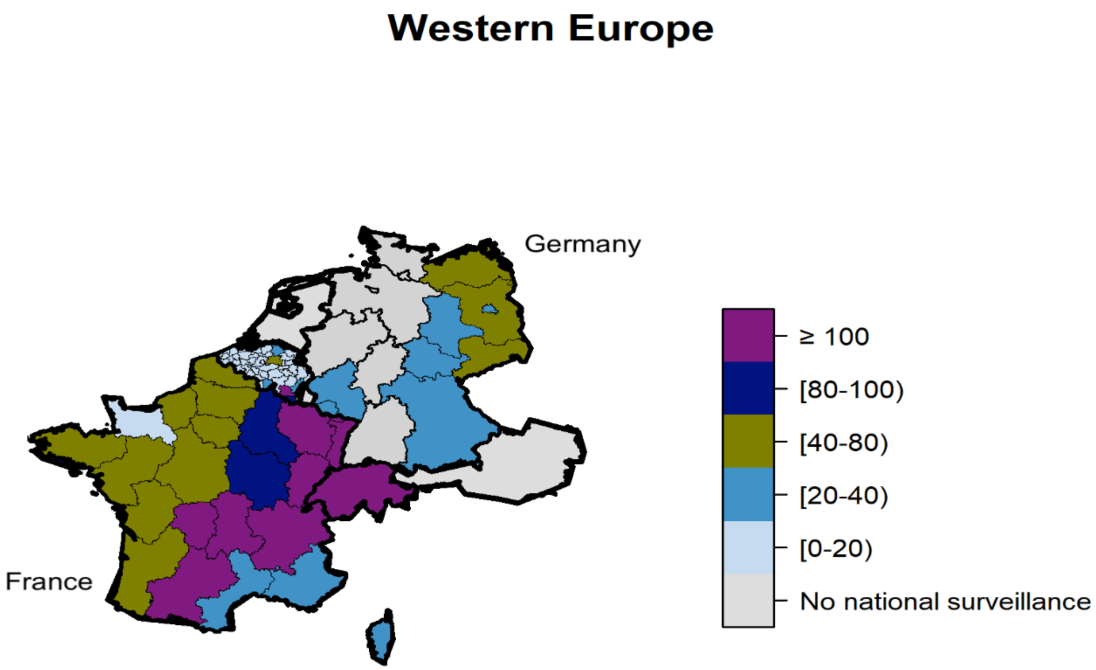

LB, Lyme borreliosis; PPY, population per year.
[a]Weighted mean for the most recent 3-year period available (all case definitions).
